# MPTHub: An Open-Source Software for Characterizing the Transport of Particles in Biorelevant Media

**DOI:** 10.3390/nano12111899

**Published:** 2022-06-01

**Authors:** Leandro Gabriel, Helena Almeida, Marta Avelar, Bruno Sarmento, José das Neves

**Affiliations:** 1 i3S—Instituto de Investigação e Inovação em Saúde, Universidade do Porto, 4200-135 Porto, Portugal; lassisg@protonmail.com (L.G.); helena.almeida@i3s.up.pt (H.A.); mavelar@i3s.up.pt (M.A.); bruno.sarmento@i3s.up.pt (B.S.); 2 INEB—Instituto de Engenharia Biomédica, Universidade do Porto, 4200-135 Porto, Portugal; 3 FEUP—Faculdade de Engenharia, Universidade do Porto, 4200-465 Porto, Portugal; 4 ICBAS—Instituto de Ciências Biomédicas Abel Salazar, Universidade do Porto, 4050-313 Porto, Portugal; 5 IUCS—Instituto Universitário de Ciências da Saúde, CESPU, 4585-116 Gandra, Portugal

**Keywords:** biological transport, mucosal delivery, mucus, particle tracking, nanomedicine, nanotechnology, software design

## Abstract

The study of particle transport in different environments plays an essential role in understanding interactions with humans and other living organisms. Importantly, obtained data can be directly used for multiple applications in fields such as fundamental biology, toxicology, or medicine. Particle movement in biorelevant media can be readily monitored using microscopy and converted into time-resolved trajectories using freely available tracking software. However, translation into tangible and meaningful parameters is time consuming and not always intuitive. We developed new software—MPTHub—as an open-access, standalone, user-friendly tool for the rapid and reliable analysis of particle trajectories extracted from video microscopy. The software was programmed using Python and allowed to import and analyze trajectory data, as well as to export relevant data such as individual and ensemble time-averaged mean square displacements and effective diffusivity, and anomalous transport exponent. Data processing was reliable, fast (total processing time of less than 10 s), and required minimal memory resources (up to a maximum of around 150 MB in random access memory). Demonstration of software applicability was conducted by studying the transport of different polystyrene nanoparticles (100–200 nm) in mucus surrogates. Overall, MPTHub represents a freely available software tool that can be used even by inexperienced users for studying the transport of particles in biorelevant media.

## 1. Introduction

Nanotechnology and materials science have come a long way over the last few decades, contributing not only to major scientific breakthroughs but also to tangible everyday solutions that are flooding the global market [1]. Consequently, humans are increasingly exposed to nanomaterials, either intentionally (e.g., in case of nanomedicine use) or inadvertently (e.g., resulting from occupational contact with nanopollutants). Outcomes of such events are dependent on complex interactions established at key gateways of the human body, namely mucosal sites, which need to be thoroughly recognized and studied [2]. Transport across different natural barriers and media is of particular importance as it largely determines the fate of nanomaterials. Indeed, the possibility of studying the motion of molecules and supramolecular structures at the nano- and microscales opened new doors to understanding the dynamics and interactions of such entities in complex biological environments [3,4,5,6]. Methods for tracking particle movement can generally fit into one of the following two categories: (i) methods that measure the ensemble average dynamics of particles, such as fluorescence recovery after photobleaching (FRAP), fluorescence correlation spectroscopy (FCS), or dynamic light scattering (DLS), and (ii) methods that can discriminate single particle movement [7]. Despite enhanced signal resulting from the bulk emission combined with the high temporal resolution provided by ensemble methods, these are known for overlooking important information regarding heterogeneous particle populations or media. Multiple particle tracking (MPT) is a well-established technique that enables tracing individual molecules, particulates, or even microorganisms at a high spatiotemporal resolution in complex biological systems such as the intracellular environment, extracellular matrix, or biological fluids (e.g., blood or mucus) [8]. In the particular case of nanomedicine, MPT allowed an understanding of the transport of nanocarriers in biological media and helped devise engineering strategies that could be useful in overcoming natural barriers to drug delivery and, thus, enhance therapeutic outcomes [9,10,11,12]. The usefulness of this powerful technique extends well beyond nanotechnology and—still in the biophysical and biomedical fields—has also been widely employed to assess the intracellular transport and kinetics of proteins, lipids, and molecular motors [13], to study the cell migration and mobility of pathogens [14,15], to determine the mechanical properties of isolated nuclei in living cells [16], or to characterize the microstructure and rheological profile of mucus and other viscoelastic fluids [17,18,19], to name only a few applications.

Details on principles, procedures, and even equipment for conducting MPT experiments are quite simple and readily accessible [8]. The processing cascade of MPT includes the acquisition of real-time videos of typically fluorescently labeled molecules or structures in biorelevant media using a microscope and a high sensitivity camera. Different processing algorithms available as plugins in common image processing programs can be employed to analyze videos and identify particles, plot their frame-by-frame coordinates, and define individual trajectories [20]. For example, the MosaicSuite package [21], NanoTrackJ [22], and TrackMate [23] for ImageJ/Fiji are popular and free-to-use plugins. Quantitative raw data in the form of biologically relevant parameters can then be extracted from obtained trajectories, either to understand the diffusive behavior of particles or to characterize the 3D network and rheological properties of the specimen in which these are embedded. While the ability to analyze motion at the single-particle level provides a valuable body of information that is not possible to attain with purely ensemble techniques, manual handling of generated large datasets constitutes a time-consuming and prone to error process [24]. Various available software has been shown useful in aiding this task [25,26,27,28,29,30]. However, these solutions offer limited possibilities for data processing customization, need intermediate to advanced computing skills for installing and use, or require payment of a license (self or that of an ancillary software such as MATLAB). Software provided with analytical equipment can also undertake MPT analysis (e.g., NIS Elements from NIKON or NTA software from Malvern), although their use is limited to specific hardware [31,32]. Similar generic open-source instrumentation software is available, but the number of compatible equipment, namely microscopes, is still restricted [33].

This work details the development of open access, standalone, user-friendly software named MPTHub for the rapid and reliable analysis of particle trajectories extracted from video microscopy. The source code and latest version of the software are available for download at https://github.com/lassisg/mpthub (last accessed date: 10 April 2022). Users can directly provide feedback on the code on the GitHub platform or via e-mail to the authors. The current version of MPTHub (v. 1.0.3) is available under Git version control as open-source software for Microsoft Windows (GNU General Public License v. 3.0). The relevance of the software was further demonstrated by characterizing the transport of 100–200 nm fluorescent polystyrene nanoparticles (NPs) in intestinal mucus surrogates.

## 2. Materials and Methods

### 2.1. Materials

Red fluorescent carboxylate-modified polystyrene (COOH-PS) NPs with nominal mean diameter values of 100 nm or 200 nm were purchased from Molecular Probes (Eugene, OR, USA). Phosphate buffered saline (PBS) tablets and purified type II mucin from porcine stomach were purchased from Sigma (St. Louis, MO, USA), and 1.5 × 1.6 cm gene frames (65 µL) were from Fisher Scientific (Hampton, NH, USA), and Amicon Ultra-0.5 mL (100 kDa MWCO) centrifugal filters were from Merck Millipore (Burlington, MA, USA). Poloxamer 407—a poly(ethylene glycol)-poly(propylene glycol)-poly(ethylene glycol) (PEG-PPG-PEG; average MW of 9.8 to 14.6 kDa)—was a kind offer from BASF (Ludwigshafen, Germany). All reagents and materials were of analytical grade or equivalent.

### 2.2. Software Development, Data Processing, and Performance

MPTHub was developed using Python (v. 3.9.5, available at https://www.python.org/; accessed on 15 September 2021) to enable researchers to easily and rapidly perform particle tracking analysis without compromising the reliability of generated results, as often associated with manual handling of large scientific datasets [34]. A few prevalidated packages were also used for the development of MPTHub, namely PySide6 for Graphical User Interface (GUI; v. 6.1.1, available at https://pypi.org/project/PySide6/; accessed on 15 September 2021), Pandas for data manipulation (v. 1.2.4, available at https://pandas.pydata.org/; accessed on 15 September 2021), Numpy for mathematical operations (v. 1.20.3, available at https://numpy.org/; accessed on 15 September 2021), MatplotLib for plotting (v. 3.4.2, available at https://matplotlib.org/; accessed on 15 September 2021), XlsxWriter for spreadsheet generation (v. 1.4.3, available at https://pypi.org/project/XlsxWriter/; accessed on 15 September 2021) and TrackPy for particle tracking analysis (v. 0.5.0, available at https://soft-matter.github.io/trackpy/v0.5.0/; accessed on 15 September 2021) [35].

**Figure 1:**
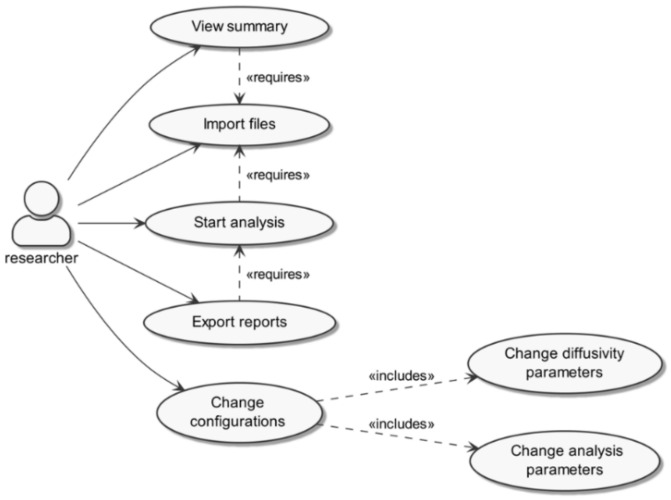
MPTHub Use Case diagram.

The software was designed to comply with three major sequential tasks: (i) import data extracted from microscopy video files, (ii) analyze data, and (iii) export results. The possibility of adjusting configurations and proceeding with reanalysis was also considered. Input comma-separated value (CSV) files were generated from microscopy videos and included data for two-dimensional Cartesian coordinates per frame (see Section 2.3.3 for details on file acquisition and preprocessing). MPTHub requires minimal initial configuration settings by the user (e.g., metric to digital units conversion) and provides several default values that can be changed. These include particle size, trajectory length cutoff (minimum number of consecutive frames that define valid trajectories), the total length of the video in frames, pixel and temporal resolution, lag time or time scale (τ) for analysis, and sample temperature during video acquisition. The software was developed in order to allow users to interact and define actions performed at multiple points of data processing (Figure 1).

Upon analysis, the software was designed to allow calculating the following parameters: (i) time-averaged mean square displacements (MSD or $\langle \Delta r^{2}\left(\tau \right)\rangle$), (ii) time-dependent diffusion coefficient or effective diffusivity (*D*_eff_), (iii) *D_w_*/<*D*_eff_> ratio, and (iv) anomalous transport exponent (*α*). MSD values for individual trajectories were determined using the following equation:

(1) $$\langle \Delta r^{2}\left(\tau \right)\rangle ={\left[x\left(t+\tau \right)-x\left(t\right)\right]}^{2}+{\left[y\left(t+\tau \right)-y\left(t\right)\right]}^{2}$$

where x and y denote coordinates between consecutive τ intervals [36]. For a particle diffusing in a simple viscous liquid, the MSD was related to *D*_eff_ according to:

(2) $$D_{\mathrm{eff}}=\frac{\langle \Delta r^{2}\left(\tau \right)\rangle }{2n\tau }$$

where n refers to dimensionality. Although data acquisition is performed in a two-dimensional plane (n = 2), if the environment is considered locally isotropic, such as in the case of mucus, then the displacements in the *x*, *y*, and *z* axes are presumably uncorrelated, and the 3D diffusion coefficient is the same as in 2D [36]. Ensemble averages of MSD (<MSD>) and *D*_eff_ (<*D*_eff_ >) were further calculated and plotted against lag time. The 〈*D*_eff_〉 at a given lag time can be compared with the theoretical diffusion coefficient of spherical particles in water (*D_w_*), as calculated by the Stokes–Einstein equation:

(3) $$D_{w}=\frac{k_{B}T}{6\pi \eta r}$$

where $k_{B}$ is the Boltzmann’s constant, *T* the temperature, η the fluid viscosity, and r the particle hydrodynamic radius. The ratio between $D_{w}$ and *D*_eff_ provides a relative measure of transport impairment of particles in a specific medium compared with water. In many cases, the diffusion of particles follows a power law scaling that allows determining α according to:

(4) $$\langle \Delta r^{2}\left(\tau \right)\rangle =4D_{0}\tau^{\alpha }$$

where *D*_0_ is the time-independent diffusion coefficient. This equation can be further completed with the inclusion of a parameter that quantifies independent noise associated with experimental measurements [37]. Additionally, the value of *α* can be used to classify particles as immobile (typically *α* < 0.2), subdiffusive (0.2 ≤ *α* < 0.9), diffusive (0.9 ≤ *α* < 1.2), or active (*α* ≥ 1.2) [32,38,39]. These ranges are empirical and can be defined otherwise; MPTHub allows users to set different values of *α* for the purpose of classifying the transport mode of NPs. The classification as diffusive corresponds to Brownian motion as predicted by the Stokes–Einstein relation [36].

The performance of MPTHub was also assessed using different sets of input files (two or four files) featuring a variable number of valid trajectories (100, 500, or 1100). Evaluated performance parameters included computing time and memory usage (defined as random access memory (RAM) peak value achieved during processing) for a full cycle of analysis, which included data import, data analysis, and results export. Testing was performed in triplicate using different hardware for replicates (details of each machine are presented in Supplementary Materials, Table S1).

### 2.3. Application of MPTHub

#### 2.3.1. Processing of Polystyrene Nanoparticles

Fluorescent COOH-PS NPs were washed twice with ultrapure water using centrifugal filters (4300 rpm, 10 min) in order to remove traces of sodium azide. NPs were then resuspended in the same solvent at concentrations varying between 5 and 20 mg × mL^−1^, and dispersions were stored at 4 °C until further use. Coating with poloxamer 407 was achieved by incubating 100 nm NPs in a 1% (*w/v*) polymer solution overnight. NPs were then washed thrice before usage, as detailed above. The hydrodynamic diameter, polydispersity index (PdI), and zeta potential were determined by dynamic light scattering and laser Doppler electrophoresis using a Zetasizer Nano ZS (Malvern Instruments, Malvern, UK). NPs were diluted between 0.1 and 0.01 mg × mL^−1^ in 10 mM sodium chloride (pH 5.8), and measurements were carried out at 25 °C.

#### 2.3.2. Preparation of Mucus Surrogates

An intestinal mucus surrogate was prepared by modifying a previously described recipe [38]: mucin was reconstituted in isotonic PBS (pH 7.4) at 30 mg × mL^−1^ or 50 mg × mL^−1^ with the aid of a vortex mixer. The mucus simulant was left to equilibrate for 30 min at room temperature before 65 µL being gently transferred into a frame chamber mounted on a glass microscope slide. Two microliters of NPs dispersed in PBS were added on top of the mucus surrogate and left to equilibrate for 1 h at room temperature after sealing the chamber with a coverslip. The final concentration of NPs in the surrogate was 0.9−7.5 × 10^−5^% (*w*/*v*) for 100 nm and 200 nm NPs.

#### 2.3.3. Microscope Configuration and Video Acquisition

The transport of fluorescent NPs was assessed by analyzing their trajectories in either mucus surrogates or water. MPT videos were recorded using a Hamamatsu ORCA-Flash4.0 digital CMOS camera (Hamamatsu, Japan) mounted on a Leica DMI6000 inverted epifluorescence microscope (Wetzlar, Germany) equipped with a 63×/1.30 NA GLYC-immersion objective. Videos (512 × 512 pixels, 16-bit, 20-s duration) were collected with the LAS X software at a temporal and pixel resolution of 33 milliseconds and 0.159 µm, respectively. Tracking resolution of approximately 20 nm was determined by calculating displacements of NPs immobilized in a strong adhesive. Acquired video files (.lif format) were preprocessed through background subtraction using ImageJ/Fiji (v. 2.1.0, available at https://imagej.net/software/fiji/; accessed on 15 September 2021), and two-dimensional Cartesian coordinates of particle centroids were detected at subpixel resolution using the MosaicSuite 2D/3D single-particle tracking plugin developed by Sbalzarini and Koumoutsakos [21]. Data were exported as CSV files and analyzed with the MPTHub software. Other image processing plugins/software can also be used to generate tracking data files, as long as these last can be converted into CSV files similar to those generated using the MosaicSuite 2D/3D plugin. Trajectories with at least 100 consecutive frames were considered valid for transport analysis. Three independent experiments, each collecting the trajectories of a minimum of one hundred NPs, were conducted for different conditions.

## 3. Results and Discussion

### 3.1. MPTHub Development, Features, and Performance

#### 3.1.1. Software Programming and Workflow

Python was selected as a programming language because of its open access and open source status, reliability/stability, relatively fast running time and low memory consumption, reduced development time, and condensed amount of generated code compared with competing software [40]. MPTHub was designed in order to perform three main sequential tasks that can be regarded as independent operational modules with particular processing sets: (i) data import, (ii) data analysis, and (iii) results export. First, data importing starts upon the user’s command for loading one or multiple input CSV files, which were previously generated by the MosaicSuite ImageJ plugin. Content and format compatibility is then checked upon selection of the ‘import’ command, and data are sanitized for compliance with TrackPy requirements for content organization. The software generates and displays a summary of total and valid trajectories per input file. Upon the user’s command, valid trajectories are processed using TrackPy for calculating relevant transport parameters. TrackPy is a validated and widely-used Python library that started its development from the mainstream Crocker–Grier algorithm using Interactive Data Language [41] and is being continuously updated [35]. This source algorithm also utilizes Monte Carlo simulations in order to estimate displacement errors based on particle size and to validate these values as adequate for optimal kernel support and accuracy of the position estimate. Exporting command generates and saves external reports that can be readily accessed by the user.

#### 3.1.2. Graphical User Interface and Utilization

**Figure 2:**
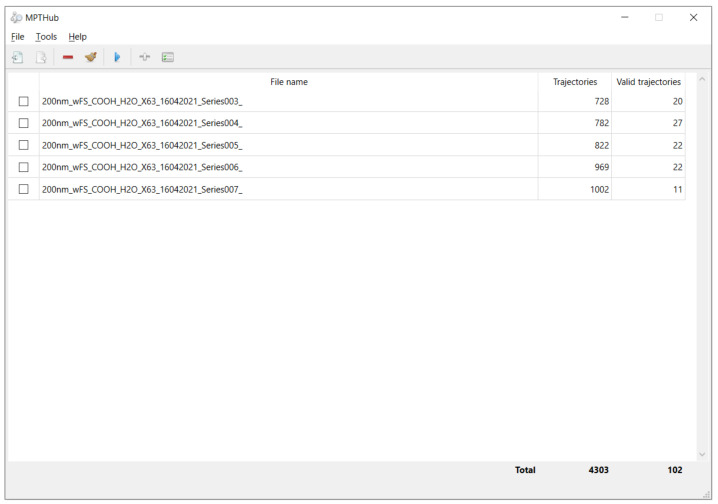
Launch screen capture of MPTHub. Multiple input files are presented (file names, total trajectories, and valid trajectories according to defined settings). Included icons are used under a CC BY 3.0 license (Copyright 2021, Yusuke Kamiyamane, available at https://p.yusukekamiyamane.com/; accessed on 15 September 2021).

**Figure 3:**
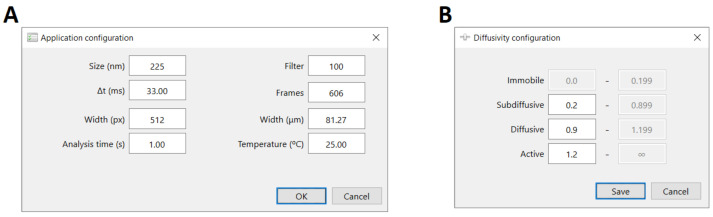
Input configuration parameters of MPTHub. Panels used by the user for defining (**A**) experimental conditions (particle size, temperature), input video properties (field width in pixels and micrometers, temporal resolution, and total length in frames), and analysis parameters (minimum number of consecutive frames needed to define valid trajectories (filter), and analysis time), and (**B**) range for the anomalous diffusion exponent in order to define transport mode.

The GUI of MPTHub features a simple, user-friendly, and flexible design, allowing intuitive use even for inexperienced individuals (Figure 2). No previous knowledge about computer programming is required, even if the source code can be easily modified by users to meet specific needs. The launch screen is divided from top to bottom into command menus, fast-access toolbars, and imported file listing and summary of total and valid trajectories. The software allows simultaneous importing and preprocessing of multiple input CSV files in order to provide a summary of total and valid trajectories. The software allows customizing configurations regarding analysis and transport mode diffusivity ranges according to specific needs (Figure 3). The user can then select which files should be analyzed, including iterations. Three data reports are generated as .xlsx format spreadsheets (named ‘Individual Particle Analysis’, ‘Transport Mode Characterization’, and ‘Stokes–Einstein Calculations’) in order to allow flexible handling of generated content according to the requirements of individual users (Supplementary Materials, Figures S1–S9). Data resulting from software analysis are only temporarily stored in local hardware unless exported by the user to a folder of choice. The last initial user input configurations are saved for convenience as a small SQLite database file (v. 3.36.0, available at https://www.sqlite.org/; accessed on 15 September 2021) that is created upon the first run of MPTHub. This embedded system does not require installation and is characterized by its portability.

#### 3.1.3. Software Performance

**Figure 4:**
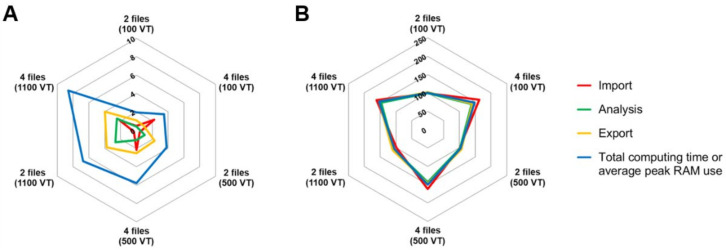
Performance analysis of MPTHub. (**A**) Computing time (in s) and (**B**) RAM peak use (in MB) when processing two or four input data files containing 100, 500, or 1100 valid trajectories (VT). Results are presented as mean values (*n* = 3).

One of the main purposes of developing MPTHub was to abbreviate the tedious and time-consuming process involved in manual data curation and processing. This usually involves the creation and use of standard template spreadsheet files, taking hours to days depending on the number of files requiring analysis and the experience of users [38,39]. Generically, MPTHub reduced the total computing time for a full cycle of data processing for multiple files (typically up to 20), including importing and exporting, to less than 10 s. In order to provide a more objective review of the computing performance MPTHub and to better understand the impact of data volume on its performance, we estimated the computing time and memory usage required for processing two sets of input files at different levels of valid trajectories (adjusted by defining suitable analysis parameters). The use of different machines (Supplementary Materials, Table S1) with various processing capabilities had minimal impact in terms of RAM peak usage, with relative standard deviation (RSD) values varying between 0.5% and 2.7% for all considered input files/valid trajectories. These values were higher for computing time values of a full cycle of data processing (RSD between 45.5% and 61.2%). In practical terms, such differences are likely irrelevant and do not limit the use of MPTHub even in less resourceful standard computers. The total processing time of MPTHub increased with higher amounts of input data (Figure 4A). Still, the maximum total processing time was only 8.6 s under unusually high input data packages (four files/1100 valid trajectories). A similar trend was noted for data analysis and results in export stages. However, import time was mostly affected by the number of input files, not valid trajectories, since processing at this stage is proportional to in-memory data storage. RAM usage was relatively constant along different processing stages and was to be mostly dependent on the number of files being processed rather than the number of valid trajectories (Figure 4B). The utilization of RAM peaked at roughly 100 MB and 150 MB when processing two or four files, respectively, from a value of around 87 MB at standby. This marginal increase and relatively stable use of memory resources throughout the import, analysis, and export stages indicate that the choice of a software design that does not store data locally had little impact on RAM usage.

### 3.2. Application of MPTHub

Demonstration of the potential of MPTHub was conducted by analyzing the motion of fluorescent COOH-PS NPs in intestinal mucus surrogates. These particles were selected because of the substantial quanta emitted upon excitation, which enables the reduction in exposure time and, therefore, potential dynamic errors. NPs were monodisperse and presented hydrodynamic values close to the nominal diameter provided by the supplier (Table 1). Changes in diameter for 100 nm NPs modified with poloxamer were negligible, while the increase in zeta potential to near neutral values suggests extensive noncovalent coating with the triblock copolymer. The formation of a dense, low MW PEG corona is known to confer mucus-penetrating properties to similarly sized NPs [42].

**Figure 5:**
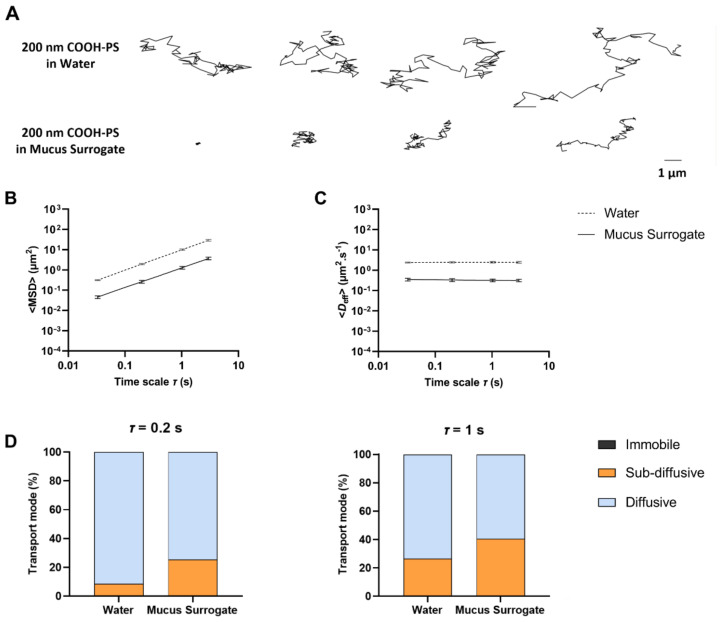
Transport behavior of 200 nm COOH-PS NPs in water or mucus surrogate containing 3% mucin. (**A**) Representative trajectories for a total duration of 3 s. Values for (**B**) <MSD> and (**C**) <*D*_eff_> of 200 nm COOH-PS NPs in water or mucus surrogate containing 3% mucin as a function of time scale. Data presented as mean ± SD (*n* = 3). (**D**) Distribution of 200 nm COOH-PS NPs in water and mucus surrogate (3% mucin) according to transport mode for *τ* = 0.2 s and *τ* = 1 s (data presented as mean values; *n* = 3).

The transport properties of NPs in different media were analyzed using the proposed software. Validation of output data was achieved by comparing with results obtained using manual processing in Microsoft^®^ Excel^®^ 2019 (v. 16.0) spreadsheets [38,39]. No differences were observed between manual and automatic data processing. We started by comparing the motion of 200 nm COOH-PS NPs in water and mucus surrogate containing 3% mucin (Figure 5A and Supplementary Videos 1 and 2). The behavior of NPs in water was random (typical of Brownian motion) and relatively constant regarding spanned length. Conversely, the transport of NPs in surrogate mucus was quite variable, ranging from highly restricted to nearly unhindered compared with water. This seems to denote a highly heterogeneous structure of the simulated medium, which is consistent with the properties of native mucus [43]. Differences in NP transport of 200 nm COOH-PS NPs were further confirmed and quantified by the ensemble (Figure 5B,C) and individual (Supplementary Materials, Figures S10 and S11) analysis of MSD and *D*_eff_. Values of <MSD> increased gradually with time scale, while those for <*D*_eff_> remained constant, thus suggesting that particles experienced thermally driven Brownian motion [36]. Also, <MSD> for 200 nm COOH-PS particles for *τ* = 1 s was almost sevenfold lower in mucus surrogate than equivalent particles in water. For the same time scale, transport in simulated mucus was nearly 6-fold lower (*D_w_*/*D*_eff_ = 5.8) than what would be expected in water, according to the Stokes–Einstein equation. The value of the *D_w_*/*D*_eff_ ratio for transport in water was 0.9, thus showing a slight deviation from the theoretical diffusivity. This could be attributable to MSD variation at longer times scales (considering the length of the trajectories) [44].

The values obtained for 200 nm COOH-PS NPs in mucus surrogate are in line with those reported by Crater and Carrier [38] using similar experimental conditions but at a longer time scale value (10 s). Nonetheless, transport hindrance of 200 nm COOH-PS NPs in either native cervicovaginal [5] or respiratory [45] mucus has been shown to be considerably higher (at least three orders of magnitude when compared with the theoretical mobility in water) than in the present work. Structural differences between native and surrogate media (obtained by simply reconstituting mucin in PBS) appear to largely justify such observations [38]. However, other possibilities may also help explain higher mobility, namely the use of a short time scale that allowed faster particles to contribute with multiple trajectories (because of their easiness of offsetting the focus plane of the microscope during video capture) [46], or the high ionic strength of the mucus surrogate that can translate into diminished effective surface charge of NPs and, thus, reduce electrostatic interactions with mucin [47]. Average values for the anomalous diffusion exponent allowed classifying the ensemble transport of 200 nm COOH-PS NPs in water as the typical pure Brownian motion in viscous fluids (*α* = 0.98) while in the mucus surrogate as marginally subdiffusive (*α* = 0.88), likely due to the establishment of mild adhesive interactions with mucin and/or increased medium viscosity [48]. Nonetheless, this value is within the range of those described for similarly sized mucus-penetrating particles in native mucus [49].

Data from individual analysis also allowed for classifying COOH-PS NPs into different classes of transport modes (Figure 5D). Virtually no particles were found to be immobile in either medium. Nearly 90% of COOH-PS NPs were found diffusive in water (*τ* = 0.2 s), contrasting with roughly 70% in mucus surrogate. Interestingly, the number of particles classified as diffusive decreased for *τ* = 1 s, particularly in water. This has been previously justified by the increase in statistical uncertainty for higher values of lag time since more minor contributions of MSDs are being considered for the calculation of its average [32]. The effect was more prominent in water since *τ* = 1 s corresponds to more than 30% of the total length of the trajectories. The promptness with which these particle transport results are generated emphasizes the usefulness of MPTHub in assessing anomalous exponent variation at different time scale values.

We further tested the applicability of MPTHub by changing experimental settings regarding the diameter (100 nm) and surface properties (PEG-modified using poloxamer 407 as coating agent) of fluorescent NPs, as well as medium composition (5% mucin). Summary data are presented in Table 2, while ensemble and individual distribution of MSD and *D*_eff_ are detailed in Supplementary Materials (Figures S12–S15). Transport of 100 nm COOH-PS NPs in mucus surrogate containing 3% mucin was only slightly reduced compared with their predictable behavior in water and still within the diffusive range (*α* = 0.91). The hindrance was increased by around 40-fold when mucin content in the medium was increased from 3% to 5%, rendering subdiffusive behavior to particles. Noncovalent PEG modification of 100 nm COOH-PS NPs impacted their mobility in both mucus surrogates, featuring enhanced transport at both 3% and 5% mucin, in line with the predicted behavior of similarly sized particles in water. These results are consistent with the established ability of densely PEG-modified NPs to confer mucus-penetrating properties [50].

## 4. Conclusions

MPTHub represents a user-friendly, free-to-use GUI-based software that allows the quantitative study of particle transport in biorelevant media. The import of tracking data, information analysis, and export of relevant results is performed rapidly and robustly, allowing individual and ensemble transport characterization of fluorescent particles in mucus surrogates. Input data can be easily generated from simple video microscopy experiments and extracted using the popular ImageJ/Fiji software, while output data are complete and allow flexible visualization and edition according to the needs of specific users. Time savings achieved with the use of MPTHub, compared with manual processing of tracking data, make it a powerful tool for high-throughput studies. However, additional work is required in order to allow further integration of the developed software with tracking algorithms (e.g., from ImageJ plugins) for direct trajectory extraction from videos, thus minimizing bias related to user input. Moreover, the software was not designed to attest to environmental isotropy, so it would be relevant to add new functionalities and increase its ability to verify this important precondition. For example, the distribution analysis of turning angles in particle trajectories can provide an indirect measure of local medium isotropy [51]. Efforts for the incorporation of other features such as statistical processing and mathematical modeling are also underway and will be included in updated versions of MPTHub. Finally, future inclusion of the MPTHub in software performance comparison iterations [20] would be interesting for a better understanding of its advantages and limitations.

## Figures and Tables

**Table 1:** Properties of COOH-PS NPs. Results are presented as mean ± SD values (*n* = 3).

NPs	Coating	Hydrodynamic Diameter (nm)	PdI	Zeta Potential (mV)
200 nm	–	225 ± 2	0.019 ± 0.011	−51.4 ± 1.8
100 nm	–	120 ± 2	0.102 ± 0.064	−42.6 ± 0.2
100 nm	Poloxamer 407	127 ± 4	0.036 ± 0.023	−4.6 ± 0.8

**Table 2:** Transport characterization of different 100 nm COOH-PS NPs in mucus surrogates containing either 3% or 5% mucin for *τ* = 1 s. Results are presented as mean ± SD values (*n* = 3).

Coating	Mucin Content (*w*/*w*%)	*D_w_*/*D*_eff_	*α*
–	3%	6.0	0.91
–	5%	250.1	0.39
Poloxamer 407	3%	1.7	0.98
Poloxamer 407	5%	4.9	0.88

## Data Availability

The data underlying this article will be shared on reasonable request to the corresponding author. The source code and latest version of MPTHub are available for download at https://github.com/lassisg/mpthub (last accessed date: 10 April 2022).

## Supplementary Materials

The following supporting information can be downloaded at: https://www.mdpi.com/article/10.3390/nano12111899/s1, Table S1: details of the three computers used for testing the performance of MPTHub, Figure S1: example of generated ‘Individual Particle Analysis’ .xlsx file spreadsheet presenting raw analysis data after processing of input CSV files, Figure S2: example of a <MSD> vs. time scale graph generated in the ‘Individual Particle Analysis’ .xlsx file, Figure S3: example of a MSD vs. time scale graph generated in the ‘Individual Particle Analysis’ .xlsx file., Figure S4: example of a <Deff> vs. time scale graph generated in the ‘Individual Particle Analysis’ .xlsx file, Figure S5: example of a Deff vs. time scale graph generated in the ‘Individual Particle Analysis’ .xlsx file, Figure S6: example of generated ‘Transport Mode Characterization’ .xlsx file spreadsheet presenting raw analysis data after processing of input CSV files, Figure S7: example of a MSD vs. time scale graph generated in the ‘Transport Mode Characterization’ .xlsx file, Figure S8: example of a spreadsheet containing individual and ensemble data summary in the ‘Transport Mode Characterization’ .xlsx file, Figure S9: example of generated ‘Stokes-Einstein Calculations’ .xlsx file spreadsheet generated after processing of input CSV files, Figure S10: individual (A) MSD and (B) *D*_eff_ of 200 nm COOH-PS NPs (≥ 100 tracked particles per experiment; *n* = 3) in water as a function of time scale, Figure S11: individual (A) MSD and (B) *D*_eff_ of 200 nm COOH-PS NPs (≥100 tracked particles per experiment; *n* = 3) in mucus surrogate containing 3% mucin as a function of time scale, Figure S12: individual (A) MSD and (B) *D*_eff_ of 100 nm COOH-PS NPs (≥100 tracked particles per experiment; *n* = 3) in mucus surrogate containing 3% mucin as a function of time scale, Figure S13: individual (A) MSD and (B) *D*_eff_ of 100 nm COOH-PS NPs (≥100 tracked particles per experiment; *n* = 3) in mucus surrogate containing 5% mucin as a function of time scale, Figure S14: individual (A) MSD and (B) *D*_eff_ of 100 nm PEG-modified COOH-PS NPs (≥100 tracked particles per experiment; *n* = 3) in mucus surrogate containing 3% mucin as a function of time scale, Figure S15: individual (A) MSD and (B) *D*_eff_ of 100 nm PEG-modified COOH-PS NPs (≥100 tracked particles per experiment; *n* = 3) in mucus surrogate containing 5% mucin as a function of time scale, Video S1: movement of fluorescent 200 nm COOH-PS NPs in water (magnification = ×63; length = 20 s), Video S2: movement of fluorescent 200 nm COOH-PS NPs in mucus surrogate containing 3% mucin (magnification = ×63; length = 20 s).
