# Supplementary material for: MPTHub: An Open-Source Software for Characterizing the Transport of Particles in Biorelevant Media
Source: Nanomaterials (Basel). 2022 Jun 1;12(11):1899. doi: 10.3390/nano12111899 (PMC9182034; doi:10.3390/nano12111899)
Supplement: Supplementary file 1 [file nanomaterials-12-01899-s001.zip › nanomaterials-1721727 supplementary material.pdf]

nanomaterials

MDPI

Supplementary Material

MPTHub: An Open-Source Software for Characterizing the Transport of Particles in Biorelevant Media

Leandro Gabriel [1,2,3,†], Helena Almeida [1,2,4,†], Marta Avelar [1,2,3,4], Bruno Sarmento [1,2,5] and José das Neves [1,2,5,*]

1 i3S—Instituto de Investigação e Inovação em Saúde, Universidade do Porto, 4200-135 Porto, Portugal; lassisg@protonmail.com (L.G.); helena.almeida@i3s.up.pt (H.A.); mavelar@i3s.up.pt (M.A.); bruno.sarmento@i3s.up.pt (B.S.)
2 INEB—Instituto de Engenharia Biomédica, Universidade do Porto, 4200-135 Porto, Portugal
3 FEUP—Faculdade de Engenharia, Universidade do Porto, 4200-465 Porto, Portugal
4 ICBAS—Instituto de Ciências Biomédicas Abel Salazar, Universidade do Porto, 4050-313 Porto, Portugal
5 IUCS—Instituto Universitário de Ciências da Saúde, CESPU, 4585-116 Gandra, Portugal
* Correspondence: j.dasneves@i3s.up.pt; Tel.: +351-220-408-800
† These authors contributed equally to this work.

Table S1. Details of the three computers used for testing the performance of MPTHub.

Computer	Properties
#1	Intel® Core™ i7-1165G7 processor (12M Cache, 2.80 GHz), 16 GB RAM
#2	Intel® Core™ i7-4500U processor (4M Cache, 2.40 GHz), 16 GB RAM
#3	Intel® Core™ i3-7020U processor (3M Cache, 2.30 GHz), 4 GB RAM

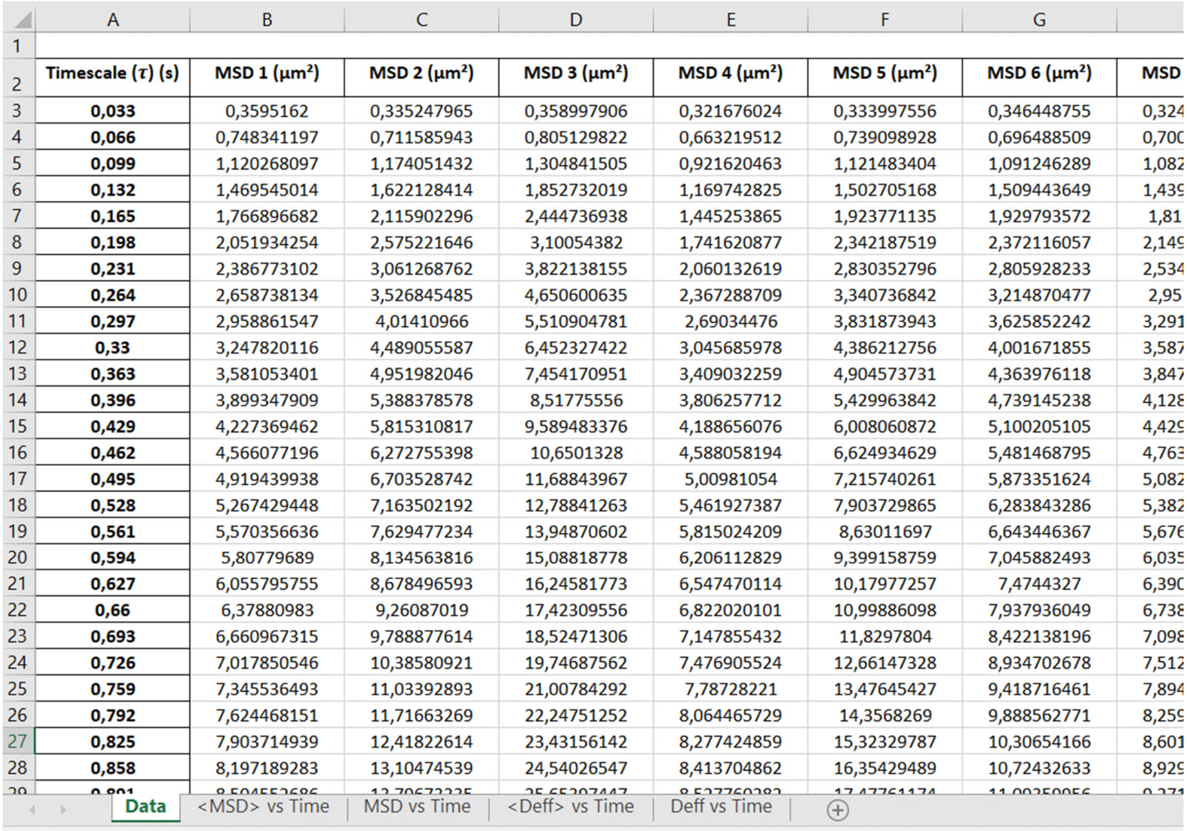

	A	B	C	D	E	F	G	H
1								
2	Timescale (τ) (s)	MSD 1 (µm²)	MSD 2 (µm²)	MSD 3 (µm²)	MSD 4 (µm²)	MSD 5 (µm²)	MSD 6 (µm²)	MSD
3	0,033	0,3595162	0,335247965	0,358997906	0,321676024	0,333997556	0,346448755	0,324
4	0,066	0,748341197	0,711585943	0,805129822	0,663219512	0,739098928	0,696488509	0,70
5	0,099	1,120268097	1,174051432	1,304841505	0,921620463	1,121483404	1,091246289	1,08
6	0,132	1,469545014	1,622128414	1,852732019	1,169742825	1,502705168	1,509443649	1,43
7	0,165	1,766896682	2,115902296	2,444736938	1,445253865	1,923771135	1,929793572	1,81
8	0,198	2,051934254	2,575221646	3,10054382	1,741620877	2,342187519	2,372116057	2,14
9	0,231	2,386773102	3,061268762	3,822138155	2,060132619	2,830352796	2,805928233	2,534
10	0,264	2,658738134	3,526845485	4,650600635	2,367288709	3,340736842	3,214870477	2,95
11	0,297	2,958861547	4,01410966	5,510904781	2,69034476	3,831873943	3,625852242	3,291
12	0,33	3,247820116	4,489055587	6,452327422	3,045685978	4,386212756	4,001671855	3,58
13	0,363	3,581053401	4,951982046	7,454170951	3,409032259	4,904573731	4,363976118	3,84
14	0,396	3,899347909	5,388378578	8,51775556	3,806257712	5,429963842	4,739145238	4,12
15	0,429	4,227369462	5,815310817	9,589483376	4,188656076	6,008060872	5,100205105	4,42
16	0,462	4,566077196	6,272755398	10,6501328	4,588058194	6,624934629	5,481468795	4,76
17	0,495	4,919439938	6,703528742	11,68843967	5,00981054	7,215740261	5,873351624	5,08
18	0,528	5,267429448	7,163502192	12,78841263	5,461927387	7,903729865	6,283843286	5,38
19	0,561	5,570356636	7,629477234	13,94870602	5,815024209	8,63011697	6,643446367	5,67
20	0,594	5,80779689	8,134563816	15,08818778	6,206112829	9,399158759	7,045882493	6,03
21	0,627	6,055795755	8,678496593	16,24581773	6,547470114	10,17977257	7,4744327	6,39
22	0,66	6,37880983	9,26087019	17,42309556	6,822020101	10,99886098	7,937936049	6,73
23	0,693	6,660967315	9,788877614	18,52471306	7,147855432	11,8297804	8,422138196	7,09
24	0,726	7,017850546	10,38580921	19,74687562	7,476905524	12,66147328	8,934702678	7,51
25	0,759	7,345536493	11,03392893	21,00784292	7,78728221	13,47645427	9,418716461	7,894
26	0,792	7,624468151	11,71663269	22,24751252	8,064465729	14,3568269	9,888562771	8,25
27	0,825	7,903714939	12,41822614	23,43156142	8,277424859	15,32329787	10,30654166	8,601
28	0,858	8,197189283	13,10474539	24,54026547	8,413704862	16,35429489	10,72432633	8,92
29	[illegible]	[illegible]	[illegible]	[illegible]	[illegible]	[illegible]	[illegible]	[illegible]

Figure S1. Example of generated 'Individual Particle Analysis' .xlsx file spreadsheet presenting raw analysis data after processing of input CSV files.

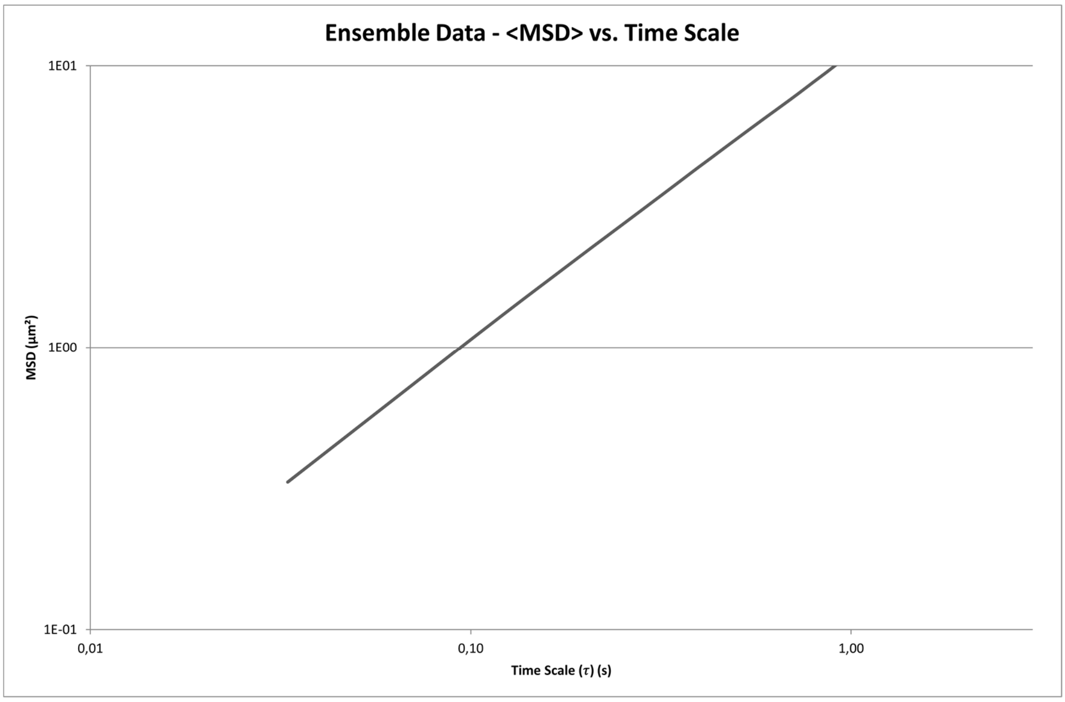

Figure S2. Example of a <MSD> vs. time scale graph generated in the 'Individual Particle Analysis' .xlsx file. Please note the logarithmic scales in both axes.

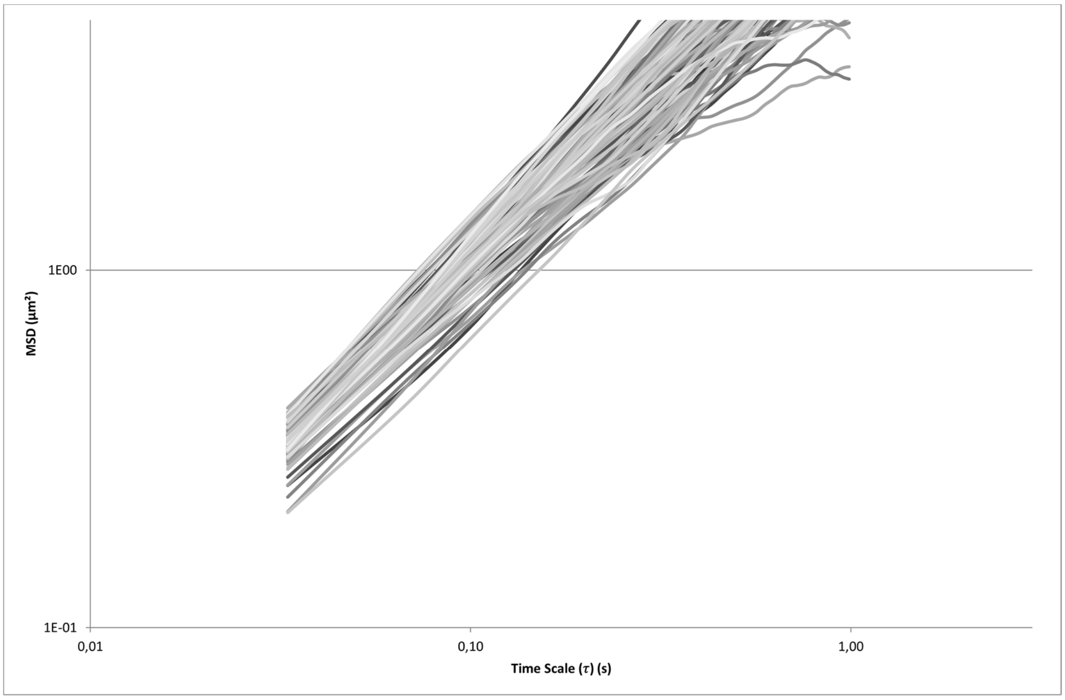

Figure S3. Example of a MSD vs. time scale graph generated in the 'Individual Particle Analysis' .xlsx file. Please note the logarithmic scales in both axes.

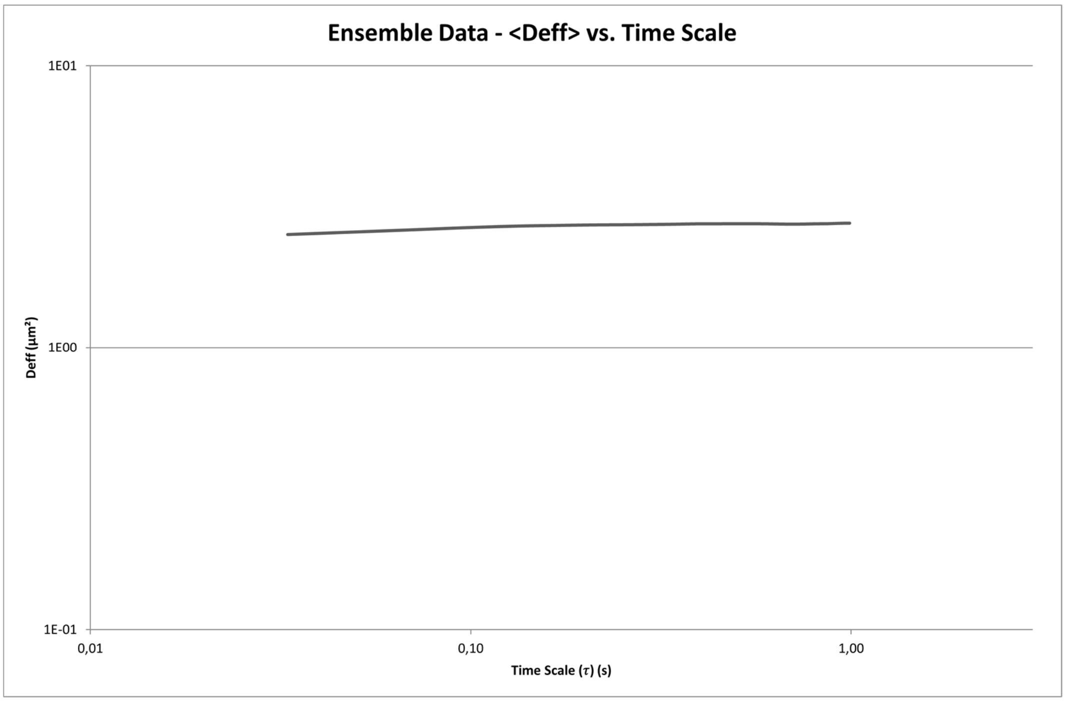

Figure S4. Example of a <D_{eff}> vs. time scale graph generated in the 'Individual Particle Analysis' .xlsx file. Please note the logarithmic scales in both axes.

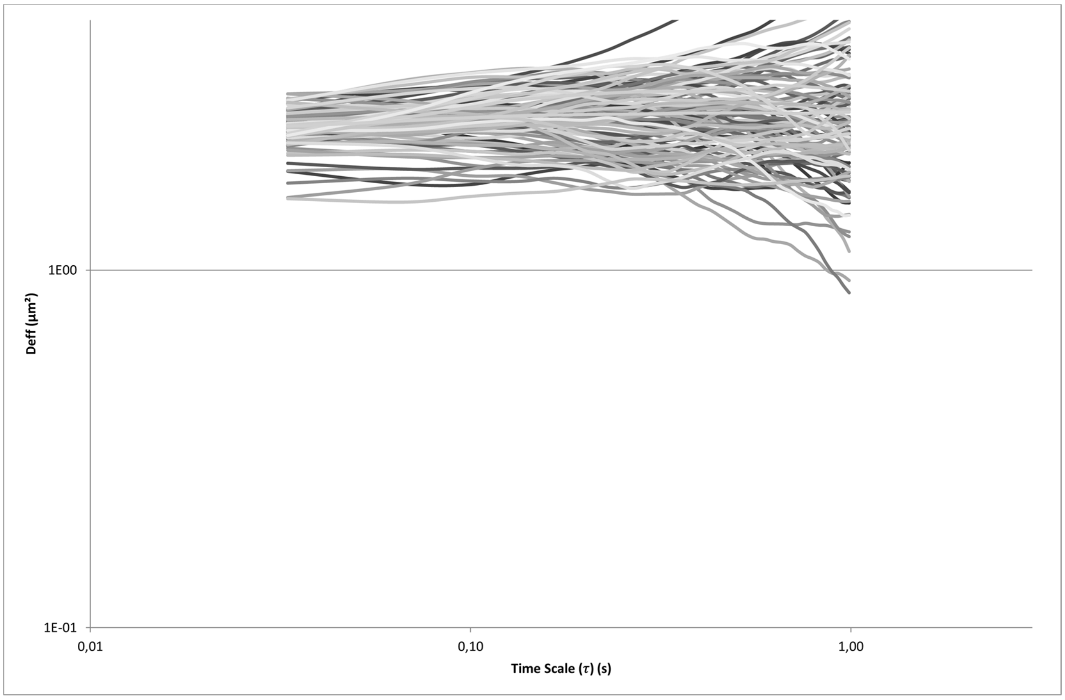

Figure S5. Example of a D_{eff} vs. time scale graph generated in the 'Individual Particle Analysis' .xlsx file. Please note the logarithmic scales in both axes.

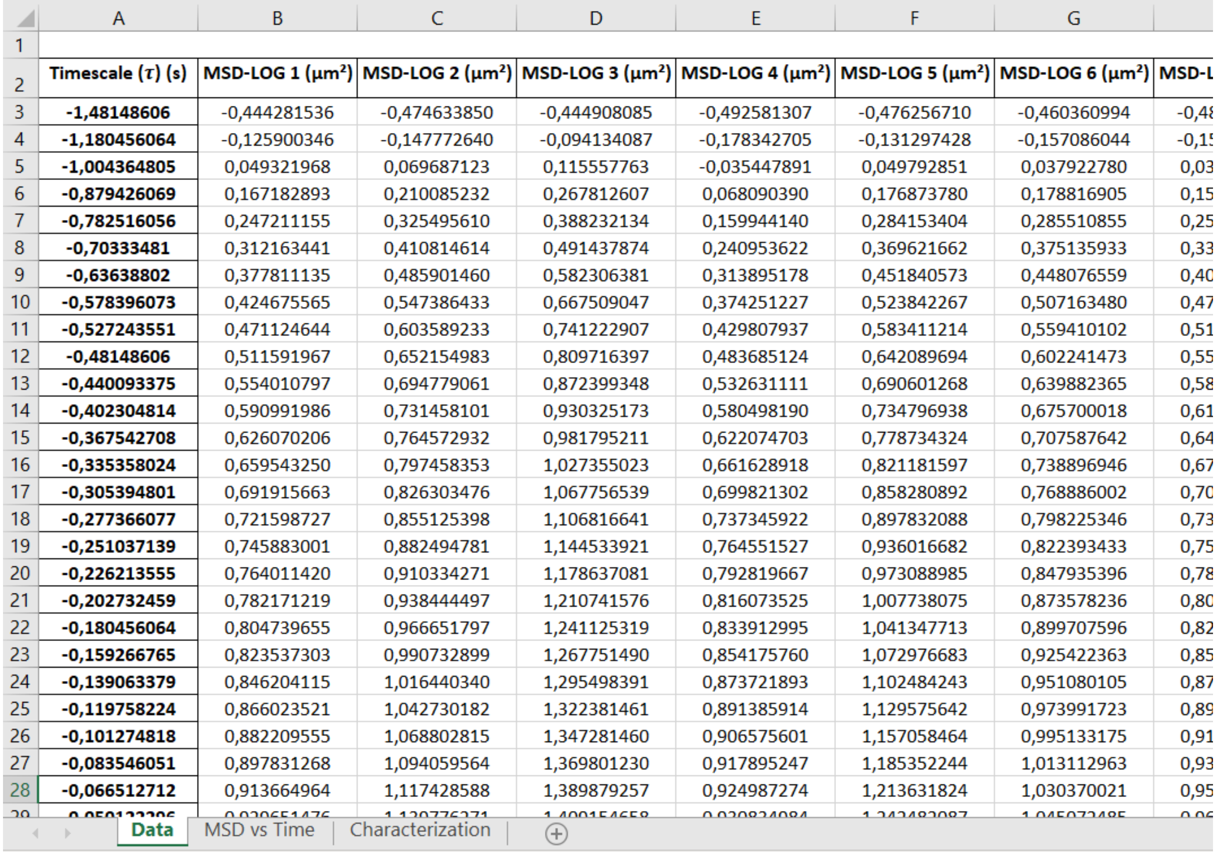

	A	B	C	D	E	F	G	
1								
2	Timescale (τ) (s)	MSD-LOG 1 (µm²)	MSD-LOG 2 (µm²)	MSD-LOG 3 (µm²)	MSD-LOG 4 (µm²)	MSD-LOG 5 (µm²)	MSD-LOG 6 (µm²)	MSD-L
3	**-1,48148606**	-0,444281536	-0,474633850	-0,444908085	-0,492581307	-0,476256710	-0,460360994	-0,4
4	**-1,180456064**	-0,125900346	-0,147772640	-0,094134087	-0,178342705	-0,131297428	-0,157086044	-0,1
5	**-1,004364805**	0,049321968	0,069687123	0,115557763	-0,035447891	0,049792851	0,037922780	0,03
6	**-0,879426069**	0,167182893	0,210085232	0,267812607	0,068090390	0,176873780	0,178816905	0,15
7	**-0,782516056**	0,247211155	0,325495610	0,388232134	0,159944140	0,284153404	0,285510855	0,25
8	**-0,70333481**	0,312163441	0,410814614	0,491437874	0,240953622	0,369621662	0,375135933	0,33
9	**-0,63638802**	0,377811135	0,485901460	0,582306381	0,313895178	0,451840573	0,448076559	0,40
10	**-0,578396073**	0,424675565	0,547386433	0,667509047	0,374251227	0,523842267	0,507163480	0,47
11	**-0,527243551**	0,471124644	0,603589233	0,741222907	0,429807937	0,583411214	0,559410102	0,51
12	**-0,48148606**	0,511591967	0,652154983	0,809716397	0,483685124	0,642089694	0,602241473	0,55
13	**-0,440093375**	0,554010797	0,694779061	0,872399348	0,532631111	0,690601268	0,639882365	0,58
14	**-0,402304814**	0,590991986	0,731458101	0,930325173	0,580498190	0,734796938	0,675700018	0,61
15	**-0,367542708**	0,626070206	0,764572932	0,981795211	0,622074703	0,778734324	0,707587642	0,64
16	**-0,335358024**	0,659543250	0,797458353	1,027355023	0,661628918	0,821181597	0,738896946	0,67
17	**-0,305394801**	0,691915663	0,826303476	1,067756539	0,699821302	0,858280892	0,768886002	0,70
18	**-0,277366077**	0,721598727	0,855125398	1,106816641	0,737345922	0,897832088	0,798225346	0,73
19	**-0,251037139**	0,745883001	0,882494781	1,144533921	0,764551527	0,936016682	0,822393433	0,75
20	**-0,226213555**	0,764011420	0,910334271	1,178637081	0,792819667	0,973088985	0,847935396	0,78
21	**-0,202732459**	0,782171219	0,938444497	1,210741576	0,816073525	1,007738075	0,873578236	0,80
22	**-0,180456064**	0,804739655	0,966651797	1,241125319	0,833912995	1,041347713	0,899707596	0,82
23	**-0,159266765**	0,823537303	0,990732899	1,267751490	0,854175760	1,072976683	0,925422363	0,85
24	**-0,139063379**	0,846204115	1,016440340	1,295498391	0,873721893	1,102484243	0,951080105	0,87
25	**-0,119758224**	0,866023521	1,042730182	1,322381461	0,891385914	1,129575642	0,973991723	0,89
26	**-0,101274818**	0,882209555	1,068802815	1,347281460	0,906575601	1,157058464	0,995133175	0,91
27	**-0,083546051**	0,897831268	1,094059564	1,369801230	0,917895247	1,185352244	1,013112963	0,93
28	**-0,066512712**	0,913664964	1,117428588	1,389879257	0,924987274	1,213631824	1,030370021	0,95
[illegible]	[illegible]	[illegible]	[illegible]	[illegible]	[illegible]	[illegible]	[illegible]	[illegible]

Figure S6. Example of generated ‘Transport Mode Characterization’ .xlsx file spreadsheet presenting raw analysis data after processing of input CSV files.

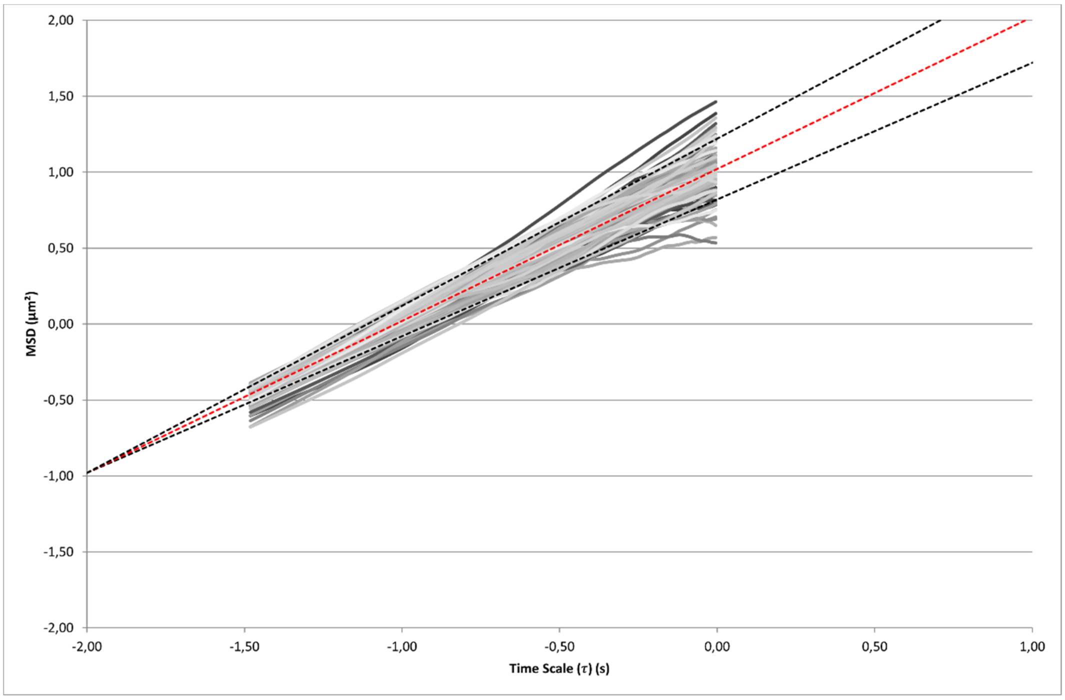

Figure S7. Example of a MSD vs. time scale graph generated in the ‘Transport Mode Characterization’ .xlsx file. Dashed lines presenting different slopes (0.9, 1.0 and 1.1) are included for guidance. Please note the linear scales in both axes.

	A	B	C	D	E	F
1	Slopes	Slopes (Excel)	R^2 (Excel)	Transport Mode	Slope	Count
2	0,946003222	0,946003222	0,999286258	Immobile	0,0-0,199	0
3	1,113317046	1,113317046	0,999057535	Sub-diffusive	0,2-0,899	19
4	1,340696417	1,340696417	0,998223359	Diffusive	0,9-1,199	76
5	1,028013443	1,028013442	0,99645975	Active	1,2+	7
6	1,225965352	1,225965352	0,994306885			
7	1,044505004	1,044505004	0,999415628			
8	0,990424520	0,99042452	0,998715094	<slope> =	0,997907128	
9	0,936409573	0,936409573	0,996765191	N =	102	
10	0,926102028	0,926102028	0,992990995	STD =	0,134222422	
11	0,820440377	0,820440377	0,979635679			
12	1,014209825	1,014209824	0,993315373			
13	0,802135139	0,802135139	0,989021957			
14	0,978573239	0,978573239	0,997081521			
15	1,040239918	1,040239918	0,992301314			
16	1,083384612	1,083384612	0,998257775			
17	0,946599831	0,946599831	0,996841926			
18	1,132902333	1,132902333	0,997483036			
19	1,061521906	1,061521906	0,991163249			
20	0,880342849	0,88034285	0,929800207			
21	1,255023774	1,255023774	0,99123038			
22	[illegible]	[illegible]	[illegible]			

Figure S8. Example of a spreadsheet containing individual and ensemble data summary in the 'Transport Mode Characterization' .xlsx file.

$$D = \frac{k_B T}{6\pi\, \eta\, r}$$

	A	B	C	D	E
5	D_W ($m^2\ s^{-1}$)	2,18110E-12	=>	D_W ($\mu m^2\ s^{-1}$)	2,1811
6	K_B (m^2 kg s^{-2} K^{-1})	13,80649E-24			
7	T (K)	298,15	<=	T (ºC)	25
8	Pi	3,1415927			
9	H_2O viscosity (Pa.s)	0,0008900			
10	Radius (m)	0,0000001	<=	Diameter (nm)	225
11	NOTE: Pa.s = kg m^{-1} s^{-1}				
13	Input cells	These cells are used to input known values			
14	Info cells	Informative cells to help understand the formula			
15	Intermediate cells	These are cells used for the calculation. Must not be changed!			
16	Output cells	Desired results are in these cells.			

Timestamp (s)	D_{eff} (μm^2-s^{-1})	D_{eff} (m^2-s^{-1})	D_W / D_{eff}
0,99000	0,003471341	3,47134E-15	628,3169

Microviscosity calculation | H2O viscosity

Figure S9. Example of generated 'Stokes-Einstein Calculations' .xlsx file spreadsheet generated after processing of input CSV files.

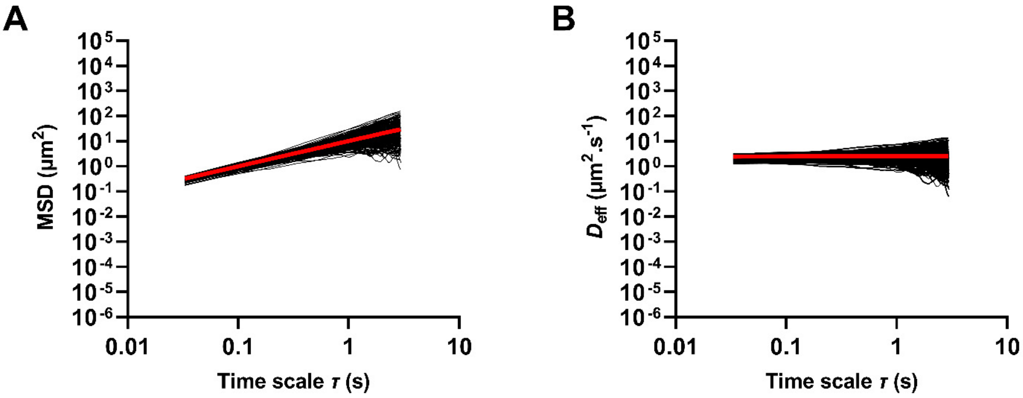

Figure S10. Individual (**A**) MSD and (**B**) D_{eff} of 200 nm COOH-PS NPs (≥ 100 tracked particles per experiment; *n* = 3) in water as a function of time scale. Ensemble values are also presented in red in both graphs.

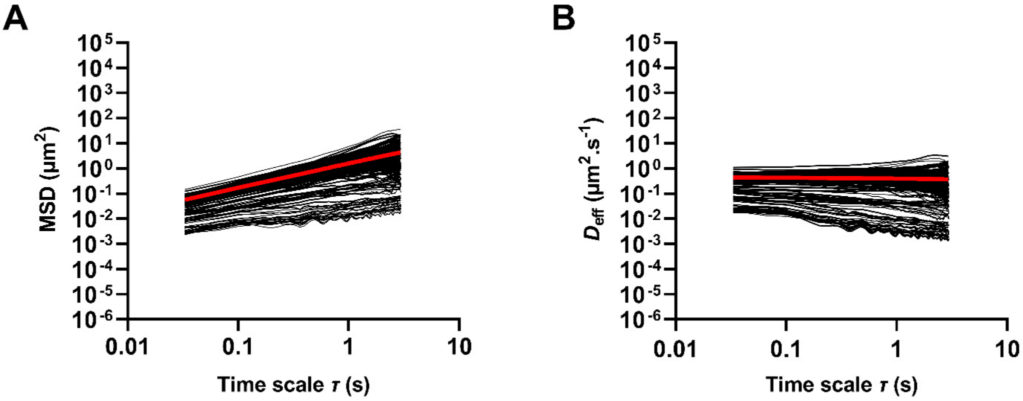

Figure S11. Individual (**A**) MSD and (**B**) D_{eff} of 200 nm COOH-PS NPs (≥ 100 tracked particles per experiment; *n* = 3) in mucus surrogate containing 3% mucin as a function of time scale. Ensemble values are also presented in red in both graphs.

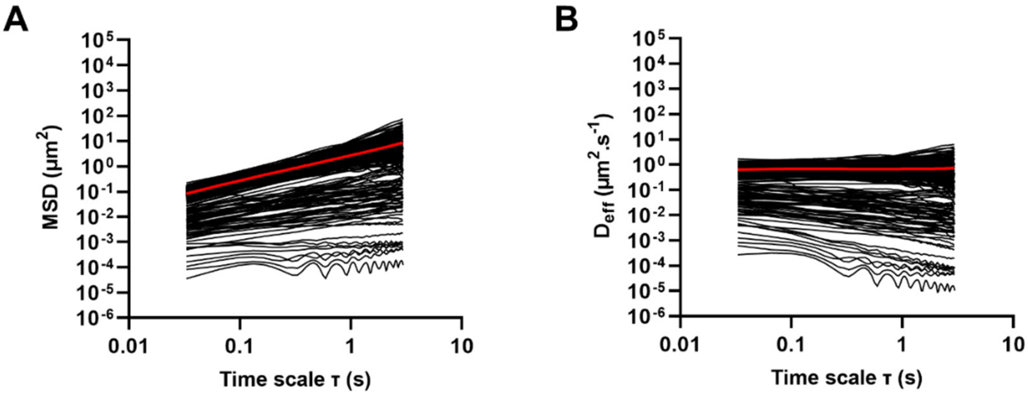

Figure S12. Individual (**A**) MSD and (**B**) D_{eff} of 100 nm COOH-PS NPs (≥ 100 tracked particles per experiment; *n* = 3) in mucus surrogate containing 3% mucin as a function of time scale. Ensemble values are also presented in red in both graphs.

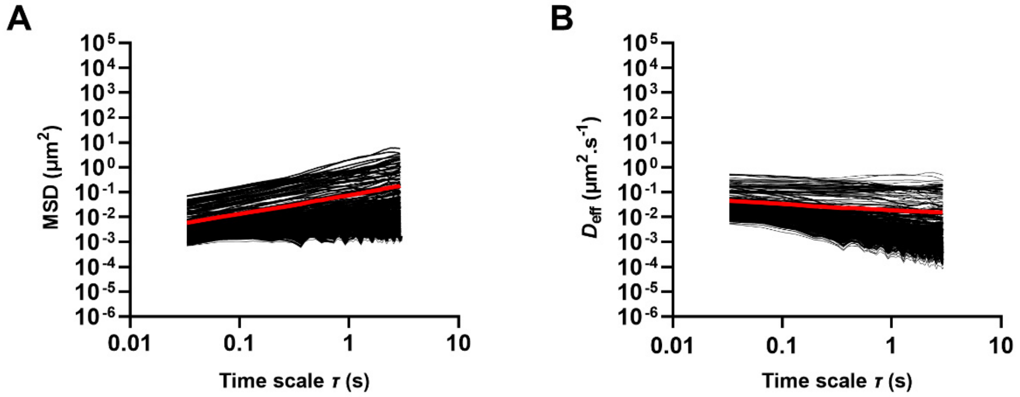

Figure S13. Individual (**A**) MSD and (**B**) D_{eff} of 100 nm COOH-PS NPs (≥ 100 tracked particles per experiment; n = 3) in mucus surrogate containing 5% mucin as a function of time scale. Ensemble values are also presented in red in both graphs.

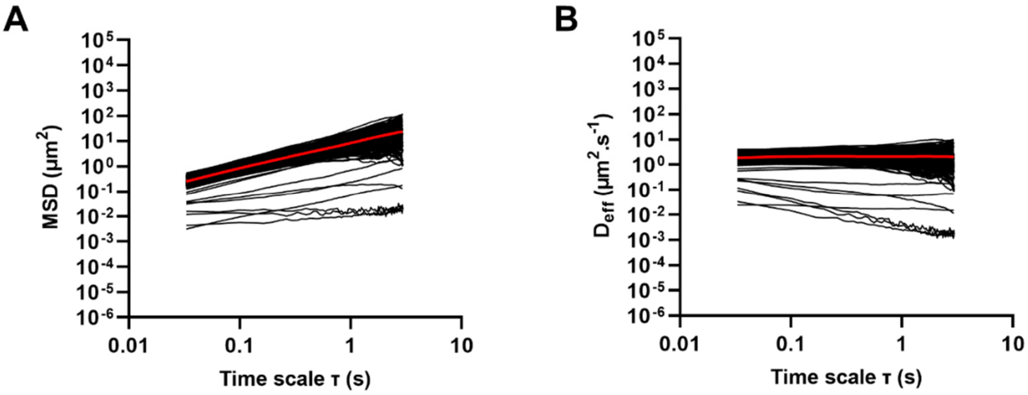

Figure S14. Individual (**A**) MSD and (**B**) D_{eff} of 100 nm PEG-modified COOH-PS NPs (≥ 100 tracked particles per experiment; n = 3) in mucus surrogate containing 3% mucin as a function of time scale. Ensemble values are also presented in red in both graphs.

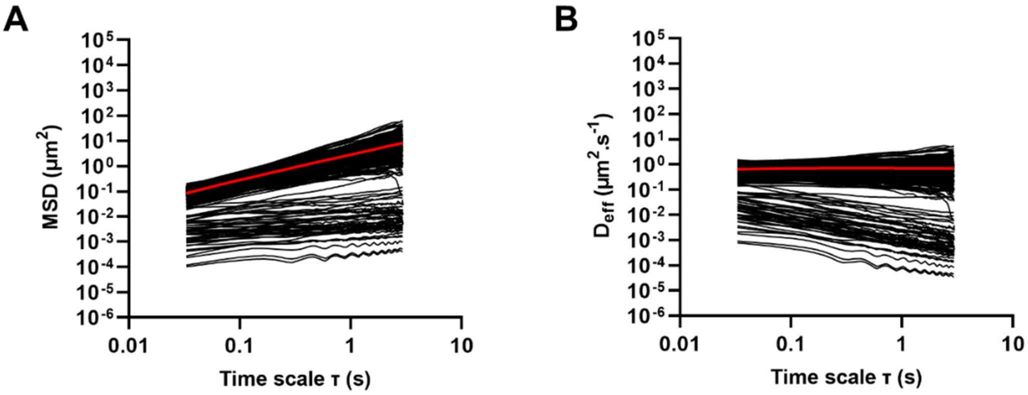

Figure S15. Individual (**A**) MSD and (**B**) D_{eff} of 100 nm PEG-modified COOH-PS NPs (≥ 100 tracked particles per experiment; n = 3) in mucus surrogate containing 5% mucin as a function of time scale. Ensemble values are also presented in red in both graphs.
